# A Criticism

**Published:** 1907-01

**Authors:** 


### EDITORIAL NOTES AND COMMENTS.

The Business Office of the JOURNAL-RECORD is 1007-1008 Century Building.

The Editorial Office is 318-319-320 The Prudential.

Address all business communications to Dr. M. B. Hutchins, Manager.

Make remittances payable to THE ATLANTA JOURNAL-REDORD OF MEDICINE.

On matters pertaining to the Editorial and Original Communications, address Dr. Bernard Wolff. Atlanta.

Reprints of original articles will be furnished at cost price. Requests for the same should always be made ON THE MANUSCRIPT.

We will present, post-paid, on request, to each contributor of an origiual article. twenty 20) marked copies of THE JOURNAL-RECORD containing such article.

### A CRITICISM.

Our contemporary, the *Mississippi Medical Monthly*, criticizes our editorial in the October issue of the Atlanta Journal-Record of Medicine but offers no solution of the problem.

"It is a matter of pride with the medical profession that its members are as a rule, and we believe it is a rule that has very few exceptions, respectable, law-abiding citizens who can be depended on to work for the uplifting and the moral welfare of their respective communities. Physicians, we speak generically, are classed among the decent people. And therefore it is a shock when we learn of a fall from decent ways on the part of any of them. Especially is this a shock when the individual is one of any prominence and is in a position that enables him by precept or example to do harm. The way of the medical editor is by no means all that might be desired, and we admit that the temptation to go crazy when the printer is calling vociferously for copy is very great, but on such occasions as the temptation overpowers him the editor should see to it that he is in the hands of those who will confine him promptly before he can influence others unduly.

"Which reflections are by way of prelude to notice of the absurdity perpetrated in the October issue of the *Atlanta Journal-Record,* a publication which is usually characterized by a strength and commonsense that make its departure all the more regretable, unless indeed the attempt be considered a burlesque.

"The stress of the recent rioting in Atlanta must have been too much for somebody's nerves, or possibly the closure of those life-saving stations known familiarly as saloons may have had a hand in it, for the leading editorial in the issue mentioned, after advocating castration instead of lynching for a certain crime, suggests that this operation be undertaken under the auspices of a ku-klux-klan. 'The identity of the criminal,' we are informed, 'could be ascertained by an orderly trial by the klan, and in this manner avoid punishing an innocent negro, as is often done in lynchings. The trial could be made more or less mysterious and impressive, for the negro stands in great awe of mysteries. An impressive trial by a ghost-like ku-klux-klan and a ghost physician or surgeon to perform the operation would make it an event the patient would never forget nor cease to enlarge upon. This would do away with the martyrdom effect of lynching as well as the demoralizing result of mob law.' And any such business would show results a thousand times more demoralizing on the members of the 'klan'—only another name for mob—than any number of ordinary lynchings, deplorable though they be. But if 'Hell's broke loose in Georgia,' to this extent and such a condition of affairs as this effusion would indicate cannot be avoided, we beg to nominate the aforesaid writer for the position of 'Lord High Executioner,' feeling sure that our candidate will win in a walk from absolute lack of opposition."

